# Reports on the Hospitals of the United Kingdom

**Published:** 1912-01-13

**Authors:** Henry Burdett


### REPORTS ON THE

### Hospitals of the United Kingdom.

By SIR HENRY BURDETT, K.C.B., K.C.V.O.

### LX.—The Hull Royal Infirmary.

This institution has existed for more than a century; it was established in 1782 and then reorganised and extended in 1887. It is interesting to note that, like other provincial institutions, as the town where it is situated has grown and prospered so the Infirmary has grown and prospered too. The work was begun in temporary premises on September 26, 1782, and a new infirmary was built and completed two years later at a cost, including the site, of £4,216. In those days people had a habit of building beyond the immediate requirements of the population, and so for nearly thirty years portions of the buildings were not occupied by patients, or even furnished. As originally constructed the building was planned as a single corridor pavilion-hospital, and consisted of one straight block with a corridor at the rear. Mr. George Pycock was the first architect, and his original plan shows a much better appreciation of sanitary and hygienic requirements than those of his successors. In 1839, 1858, and 1863 additions were made to the original buildings, which provided further accommodation but tended materially to alter for the worse the structural qualities of the buildings, having regard to the uses to which they are put. We have no particulars of the cost of these additions, which must have been considerable, but they certainly did not tend to improve the Infirmary for hospital purposes.

In 1882, at the ninety-ninth annual meeting, it was decided to celebrate the centenary, and with this object it was determined to increase the invested funds by £20,000 and the subscription list by £2,000 a year. Although the appeal which followed was only partially successful, it was soon afterwards agreed to expend £30,000 upon the purchase of additional land and the erection thereon of new buildings from the plans of Mr. Saxon Snell. The foundation-stone was laid by the Duke and Duchess of Edinburgh on October 1, 1884. The new wing was opened by the Lord Lieutenant of the East Riding (Lord Herries) at the end of 1885, when more than the £20,000 had been subscribed.

### A Point of History.

It is worthy of notice that the Chairman of the hospital, Mr. Henry Simpson, became interested in the Infirmary so long ago as 1840, when he gave as a schoolboy his first donation to the Bazaar Fund in aid of the extensions made in that year. In connection with the Duke of Edinburgh's visit the Hull General Infirmary became the Hull Royal Infirmary, by which name it is now known and widely appreciated throughout the East Riding. What inconveniences and disagreeables were put up with in hospitals thirty years ago was graphically illustrated by a member of the present staff, who told us that within his recollection, some thirty years, the service staircases were circular and the cadavers had to be removed in a shell which was carried on the back of the porter down the circular staircase to the mortuary.

### Present Condition of the Hospital.

This is a good practical work-a-day hospital without frills or anything that can be done without, that is, where its omission can be supplied by zeal, energy, and service on the part of the staff. Hence it is evident in many directions that so-called economy is so generally insisted upon by someone in authority that it verges on the maintenance of avoidable inefficiency. This fault, for it is a fault, is displayed in the absence of common and reasonable comforts for the patients in the way of furniture in the wards, where the friends of patients have practically nowhere to sit but on the beds. In several wards the appearance would be improved and efficiency increased by the supply of modern up-to-date glass-topped tables provided with the usual accommodation for dressings, bandages, and ward necessaries; by the provision of new patients' lockers throughout the hospital; of good poison-cupboards, and of a few comfortable chairs with at least one reclining-couch or ambulance trolley in each ward.

Again, it is false economy and not just either, that probationers in the third year, who are paid £17 salary inclusive, should be used as staff nurses instead of employing fully trained and certificated staff nurses. The whole nursing staff, with the exception perhaps of eleven sisters, are inadequately paid. Apart from the sisters there are no fully trained nurses employed, but the whole of the work has to be done by forty-six probationers, those in the first year receiving £8, in the second £10, in the third £17 a year. When they enter upon their fourth year the probationers are moved to the private nursing staff and they then receive £25 salary, which may be increased to £30 at the expiration of the fourth year if they continue on the private staff. The structural arrangements of the hospital make it an exceptionally difficult one to nurse and administer, and we formed the strong conviction that the nursing staff are underpaid and overworked or pressed. Thus when the hospital was reconstructed in 1887, the ward unit created consisted of one large rectangular ward with 28 beds, a circular ward of 12 beds, and three beds in private wards, making a total of 43 beds to each unit, which is in charge of a sister who has no one but probationers to help her in the work. The character of the work done in this hospital demands for efficient nursing purposes that the sister-in-charge for each unit should have under her two fully-trained certificated staff nurses with salaries of at least £25 to £30 per annum, and sufficient probationers in addition. This moderate and necessary change should be effected without delay, for it is but just to the lady superintendent, the work, and the nursing staff, and should add materially to the comfort of the patients.

The wards in themselves are good though inconvenient to administer, but the nursing arrangements, the fittings, and the furniture are not up to modern requirements. The sleeping accommodation for the domestic staff of the hospital is objectionable in the sense that there is practically no privacy for each woman employed. This fact for moral, hygienic, and humanitarian reasons demands the prompt attention of those responsible. The wards have been provided with excellent verandas, which form one of the most satisfactory and best features of this hospital. When we visited it in 1893 "we were much pleased with the then management, and found that the structural imperfections were largely neutralised by the zeal and energy of the resident staff" at that date. We formed exactly the same impression during our inspection of this hospital on September 7, 1911, a wonderful proof of the exceptionally good service rendered to the Committee by Miss Binns and the staff.

### The Changes Needed.

It demonstrates the high personal standard of work which every worker maintains throughout the establishment. We hope, therefore, that the Managing Committee will contribute their part to the well-being of this important hospital by at once deciding to pay adequately the nursing staff and to strengthen it by the addition of a sufficient number of fully-trained staff nurses. We hope further that the Committee will agree to remove the deficiencies in the present furnishing, fittings, and bedding where the use of flock should be forbidden in view of the recent reports issued by the Local Government Board. It is satisfactory to note that the bedding is maintained in as high a state of efficiency as possible under a contract which provides that every mattress shall be taken to pieces, disinfected, cleansed, and refilled after use. This is in striking contrast to the inefficiency in this respect of the arrangements we found to prevail at the York County Hospital.

### The Laundry and Mortuary.

It would be an economy and advantage in many ways to rearrange the present laundry. It is sadly deficient in the sense that it has no separate foul-linen section. By reorganisation it should be possible to keep the linen of the staff wholly distinct and separate from that of the hospital proper—an arrangement which should materially improve the appearance and get-up of the various articles. For hygienic reasons alone these two changes are urgently called for. Indeed, we believe that a thorough reorganisation of the whole laundry would greatly add to its efficiency and improve the quality of the work at present done there. The hospital is up to date in the sense that it has a sufficient mortuary unit which includes the provision of a jury room, a coroner's court room, a mortuary, and a post-mortem room. The atmosphere of the latter when we inspected it was so bad, and indeed offensive, though it was empty, as to prove that it urgently requires re-ventilation if due regard is to be paid to the health of the staff and the efficiency of the work that must be done there.

### Congratulations to the Secretary.

Mr. Benjamin Brooks has held the office of secretary to this hospital for upwards of thirty years. He looks as active, hale, and debonair as a young man of half his age, and is full of zeal in the cause of the hospital. This is a striking testimony to the fact that continuous work for a great hospital zealously pursued for the best part of a lifetime may prove one of the healthiest as it is one of the very best occupations for a real man and true gentleman. We should judge Mr. Brooks to possess the best attributes of a keen Yorkshireman, and he is deservedly held in universal esteem and honour. The total receipts, including legacies, were £20,330 in 1910, when they exceeded the total ordinary expenditure by about £4,500—a proof of Mr. Brooks' energetic and successful work. We could wish that he would keep the accounts upon the Uniform System, for then full justice might be done to the economical administration of this hospital and to the working-class contributors especially, who gave almost as much money in 1910 as the total contributors of all other classes included under the headings of annual subscriptions, benefactions, donations, and collections in places of worship. As the accounts are at present rendered it is only after a careful examination of the receipts that the contributions of the working classes can be identified, for they appear wrongly under the heading "benefactions and donations," from which they should be kept wholly distinct in justice to the givers.

A pleasing feature of the accounts is that the clerks and assistants of Hull gave £312 to the Hull Royal Infirmary in 1910. Hull contains a population of quite two hundred thousand people, and it seems to us that as a city it would gain much by the presence of one of the prophets of old to conduct a crusade in favour of personal service on the part of all classes of the citizens in the days of health in the cause of the sick. A small annual subscription list of under £3,000 a year and a contribution of only £1,286 from so large and prosperous a community as that of Hull, with probably over two hundred thousand inhabitants, is sad reading for those who have an accurate knowledge of what other cities and towns have done, in proportion to their population, for their great voluntary hospitals.

Whilst on the subject of finance it may be well to record, in view of what we have said above, that the Hull Royal Infirmary made a profit of upwards of £350 out of the earnings of the private nurses' fund in 1910, after meeting the whole of its ordinary and extraordinary expenditure.

